# Synthesis of Marine Cyclopeptide Galaxamide Analogues as Potential Anticancer Agents

**DOI:** 10.3390/md20030158

**Published:** 2022-02-22

**Authors:** Daichun Li, Xiaojian Liao, Shenghui Zhong, Bingxin Zhao, Shihai Xu

**Affiliations:** Department of Chemistry, College of Chemistry and Materials Science, Jinan University, Guangzhou 510632, China; ldcman1733061011@stu2017.jnu.edu.cn (D.L.); tliaoxj@jnu.edu.cn (X.L.); zhongsh@stu2014.jnu.edu.cn (S.Z.)

**Keywords:** drug design, anticancer, apoptosis, cyclic pentapeptide, galaxamide analogues

## Abstract

In this paper, eight new galaxamide analogues (**Z-1**~**Z-8**) were synthesized and evaluated for their cytotoxic activities against five cancer cell lines, MCF-7, MD-MBA-231, HepG2, Hela, and A549, using MTT assays. The modified analogue **Z-6** displayed broad spectrum cytotoxic activity toward each tested cell line with IC_50_ values of 1.65 ± 0.30 (MCF-7), 2.91 ± 0.17 (HepG2), 4.59 ± 0.27 (MD-MBA-231), 5.69 ± 0.37 (Hela), and 5.96 ± 0.41 (A549) μg/mL, respectively. The galaxamides **Z-3** and **Z-6** induced concentration-dependent apoptosis of the MCF-7 cells after 72 h as evaluated by the flow cytometry experiment. The results showed that these compounds could induce MCF-7 cell apoptosis by arresting the G0/G1 phase of the cell cycle and finally achieving the effect of inhibiting the proliferation of MCF-7 cells.

## 1. Introduction

Cyclopeptides mainly isolated from various marine species, which exhibited antitumor, [1,2,3] antiviral, [4] anti-inflammatory [5,6] and antibacterial [7,8,9] activities. Cyclopeptides usually exhibited superior biological activities due to their conformational rigidity and resistance to proteolytic degradation. [10] Therefore, cyclopeptides had attracted great attention and become one of the important components in the development of antitumor drugs. [11] To enhance the antitumor activity of cyclopeptides, they were always modified by changing the configurations of amino acid residues. In addition, the fragment of proline was widely existing in natural cyclopeptides, [12] which might be the active group for antitumor and antibacterial activities.

**Figure 1:**
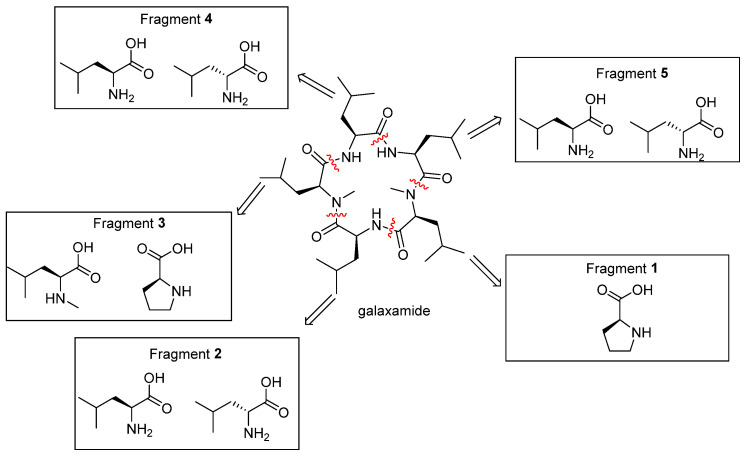
Design in the series of galaxamide analogues.

In our group, previous phytochemical investigation on the alga of *Galaxaura filamentosa* had led to the isolation of a novel cyclic pentapeptide, galaxamide (Figure 1). It was composed of five *L*-leucines including two *N*-methylated ones, which showed significant antitumor activities against GRC-1 and HepG2 cell lines. [13] In the preliminary work, the galaxamide analogues had been synthesized by replacing one of the leucines with penylalanine and changing the configuration of amino acids, which also showed potential antitumor activities. [14,15,16]

In this paper, eight new galaxamide analogues (**Z-1**~**Z-8**) were synthesized by replacing one or two *N*-Me-*L*-leucines in the galaxamide with *L*-proline or changing the stereo configuration of leucine. In vitro antitumor activities of these new compounds against MCF-7, HepG2, Hela, MD-MBA-231, A549, and HUVEC cell lines were also evaluated by MTT assay. On the one hand, a great majority of the synthesized compounds showed strong antitumor activities. On the other hand, they showed weak inhibitory effects on the non-neoplastic cells. Particularly, the IC_50_ values of **Z-3** and **Z-6** on all of the tested cancer cell lines were smaller than those of galaxamide. Furthermore, we described the effects as well as the antitumor mechanisms of **Z-3**, **Z-6,** and galaxamide as they induced apoptosis of MCF-7 cells.

## 2. Results and Discussion

### 2.1. Chemistry

**Figure 2:**
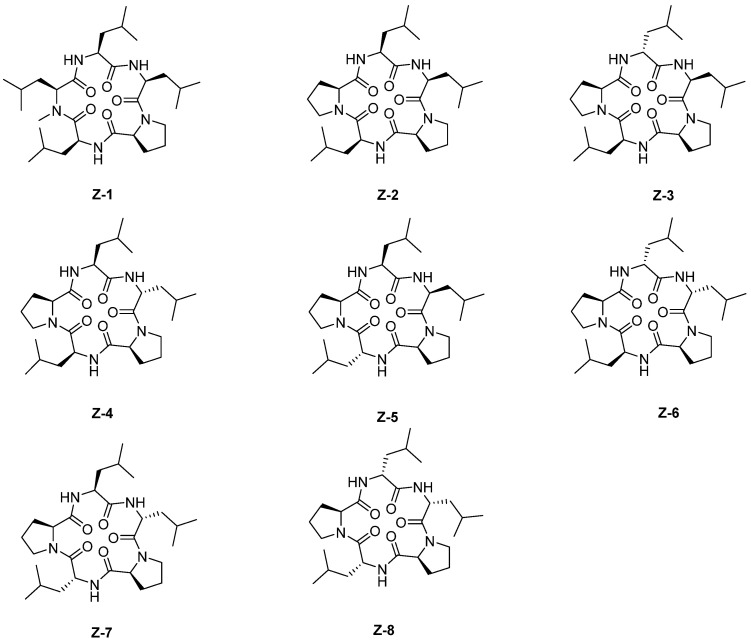
The structures of **Z**-**1**~**Z**-**8**.

On the basis of galaxamide, eight new galaxamide derivatives were designed and synthesized by replacing one or two *N*-Me-*L*-leucine with *L*-proline or changing the stereo configuration of leucine from *L* to *D* (Figure 1 and Figure 2).

**Scheme 1:**
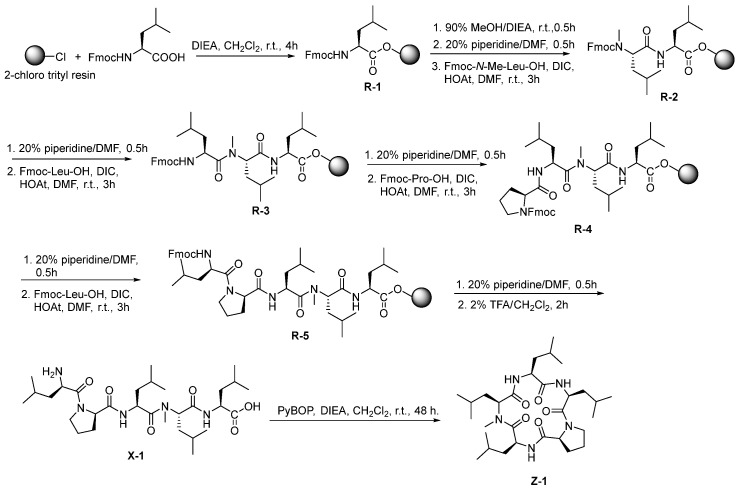
Synthesis scheme of **Z**-**1**.

**Scheme 2:**
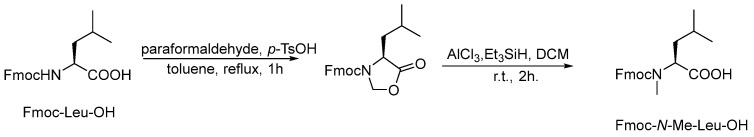
Synthesis scheme of Fmoc-*N*-Me-Leu-OH.

First, the linear pentapeptide was synthesized by using Fmoc peptide synthetic strategy with 2-chloro trityl resin. [17] Furthermore, the cyclic peptide was compounded by liquid-phase cyclization. The cyclization site of the linear pentapeptide was selected between two consecutive leucines. The detailed schematic illustration of the synthetic route was shown in Scheme 1 (taking **Z**-**1** as an example). Among them, Fmoc-*N*-Me-Leu-OH was synthesized by the oxazolidinone intermediate method (Scheme 2). [18]

### 2.2. Biological Activity

#### 2.2.1. Inhibitory Activities of Galaxamide and Its Analogues

The cytotoxicity of the synthesized compounds against five different human cancer cell lines (MCF-7, MD-MBA-231, HepG2, Hela and A459) and one type of normal human cell line (HUVEC) was investigated by MTT assay, the IC_50_ values of which are shown in Table 1. The results indicated that **Z-3** and **Z-6** exhibited stronger cytotoxic activities against each cell line than galaxamide, but that they showed weaker inhibitory effects on HUVEC. Particularly, **Z-6** had the strongest inhibition rate.

#### 2.2.2. Effects of Analogues on the Cell Cycle of MCF-7

**Figure 3:**
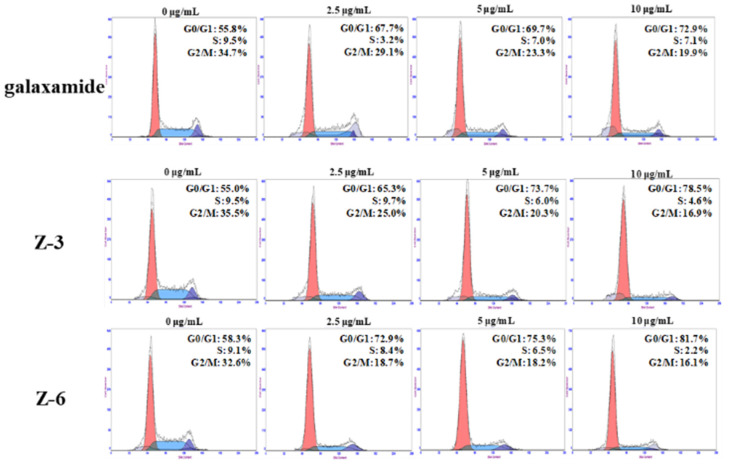
Cell cycle phase in MCF-7 induced by galaxamide, **Z-3,** and **Z**-**6** when exposed for 72 h.

Compounds **Z-3** and **Z-6** exhibited better inhibition effects on the growth of all cancer cell lines than the others, especially on that of MCF-7. Therefore, propidium iodide (PI) staining method was used to further investigate the changes in the cell cycle distribution of MCF-7. In particular, MCF-7 cells were exposed to galaxamide, **Z-3,** and **Z-6** with concentrations of 0, 2.5, 5, and 10 µg/mL for 72 h. Generally, the main effects of galaxamide, **Z-3,** and **Z-6** on MCF-7 cells were in the G0/G1 phase. There was no obvious sub diploid peak in flow cytometry. However, as the concentration increased, the number of cells in the G0/G1 phase also gradually increased. Hence, the effects of galaxamide, **Z-3,** and **Z-6** on MCF-7 cells mainly blocked the growth of cells in the G0/G1 phase (Figure 3).

#### 2.2.3. Apoptotic Mechanism Study of Galaxamide and Its Analogues

**Figure 4:**
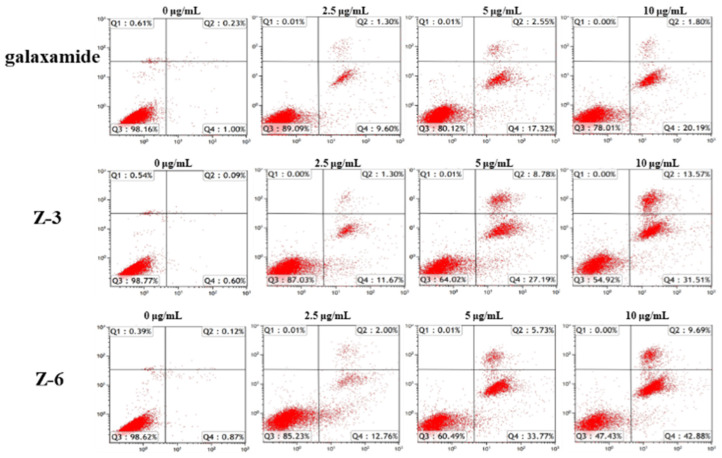
Monomers used in the synthesis of galaxamide analogues.

The Annexin V-FITC/PI double staining method was used to analyze the changes in the apoptosis of MCF-7 cells after 72 h of galaxamide, **Z**-**3,** and **Z**-**6** exposure. The results showed that the apoptosis rate of MCF-7 increased with the increasing of the concentration after 72 h of incubation. At low concentrations, the effects of all the tested compounds were not obvious. When the concentration reached 10 μg/mL, the apoptotic effects of galaxamide, **Z**-**3,** and **Z**-**6** on MCF-7 cells were more obvious in the late apoptotic state. Particularly, the apoptotic effects of **Z**-**3** and **Z**-**6** on MCF-7 cells were significantly higher than those of galaxamide (Figure 4).

#### 2.2.4. Effects of Galaxamide and Its Analogues on Cell Nuclear Integrity

**Figure 5:**
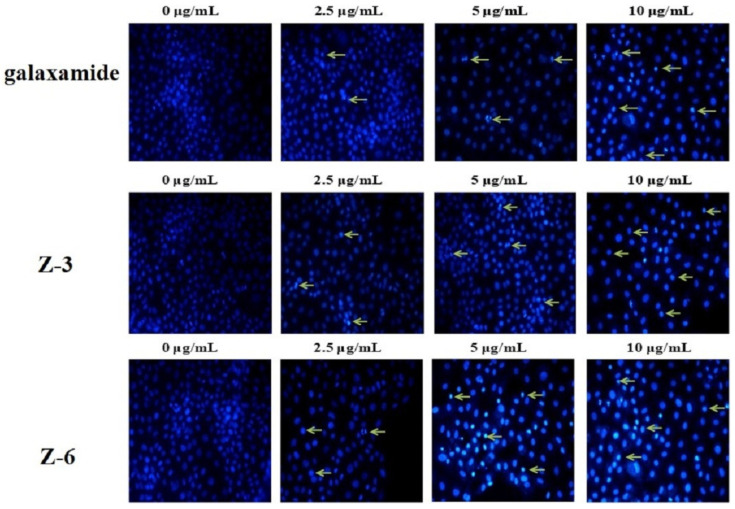
Morphological observation of galaxamide and its analogues inducing apoptosis in MCF-7 cells as indicated by Hoechst 33,342 staining.

The morphological alteration of MCF-7 cells was analyzed by the method of Hoechst 33,342 staining (Figure 5). After the MCF-7 cell line was treated with galaxamide, Z-3, and Z-6 for 72 h, the morphology of the nuclei was significantly changed. The pyknotic nuclei increased with the increasing concentration of these compounds. At the same time, karyorrhexis and karyolysis were observed, indicating that these compounds could cause obvious cell apoptosis.

## 3. Materials and Methods

### 3.1. General

NMR spectra were measured on a Bruker Avance 300 spectrometer (Bruker, Billerica, MA, USA) in CDCl_3_. Chemical shifts are reported as *δ* values in parts per million (ppm) relative to tetramethyl silane (TMS) and *J* values are expressed in Hertz. The ESI mass spectra were obtained on a LCQ DECA XP LC-MS mass spectrometer. Silica gel (200–300 mesh) for column chromatography and silica GF254 for TLC were produced by the Qingdao Marine Chemical Company (Qingdao, China). All reactions avoiding water and air were carried out under nitrogen atmosphere conditions. The starting materials and reagents used in reactions were obtained commercially from Acros, Aldrich, and GL Biochem, and used without purification, unless otherwise indicated.

### 3.2. Compounds

#### 3.2.1. Synthesis of Fmoc-N-Me-Leu-OH

A solution of Fmoc-Leu-OH (7.07 g, 2 mmol), paraformaldehyde (4.00 g), and p-toluenesulphonic acid (0.20 g, catalytic amount) in toluene (100 mL) were suspended in a 250 mL three neck RBF. The mixture was stirred and refluxed for 30 min in a Dean-Stark setup and monitored by TLC. Following this, the solution was allowed to be cooled and poured into saturated aqueous NaHCO_3_. The organic layer was dried over anhydrous sodium sulfate and concentrated, the residue was purified by column chromatography on silica gel (eluant ratio of *V_petroleum ether_/V_ethylacetate_* = 8:1) to obtain 7.15 g of white powder with a yield of 97.9%.

Then, the white powder (7.00 g, 2 mmol), AlCl_3_ (4.00 g, 3 mmol), triethylsilane (5 mL), and dry DCM (150 mL) solution were added to a 250 mL round-bottom flask. The reaction was stirred at ambient temperature until the TLC showed an absence of the starting material. The organic phase was washed with 1M HCl. Subsequently, the organic layer was dried and filtered concentrated with anhydrous sodium sulfate. It was then dried once more under a vacuum and purified using silica-gel column chromatography (eluant ratio of *V_petroleum ether_*/*V_ethyl acetate_* = 8:1). Finally, 6.54 g of white powder was obtained with a yield of 92.7%. ^1^H NMR (300 MHz, DMSO-*d*_6_), *δ*_H_: 12.77 (s, 1H), 7.90 (t, *J* = 7.5 Hz, 2H), 7.65 (td, *J* = 7.8, 3.8 Hz, 2H), 7.49–7.38 (m, 2H), 7.38–7.28 (m, 2H), 4.69–4.45 (m, 1H), 4.46–4.13 (m, 3H), 2.72 (s, 3H), 1.80–1.43 (m, 2H), 1.37 (d, *J* = 12.2 Hz, 1H), 1.00–0.60 (m, 6H); ESI-MS *m/z*: 368.2 [M + H]^+^, 385.1 [M + NH_4_]^+^, 390.1 [M + Na]^+^.

#### 3.2.2. Synthesis of Linear Pentapeptide HO-Leu-N-Me-Leu-Leu-Pro-Leu-H (X-1)

Synthesis of Fmoc-Leu-Resin (**R**-**1**). The linear peptide was synthesized by using 2-chlorotrityl chloride (CTC) resin following the standard Fmoc strategy. Firstly, using 15 mL of dichloromethane dissolved Fmoc-Leu-OH (1.06 g, 3 mmol), and then adding 0.5 mL of DIEA. Then, the liquid was added to the peptide synthesis tube containing the CTC resin (1.00 g, load: 0.985 mmol/g). The reaction tube was placed on a shaker at room temperature for reaction 4 h. After the reaction, the resin was washed with DCM (3 × 5 mL), DMF (3 × 5 mL), and MeOH (3 × 5 mL). After filtration, the remaining unreacted sites of the resin were capped with a solution of DCM/MeOH/DIEA (4:5:1, 10 mL) for 30 min. Deprotection of Fmoc group was carried out using 20% piperidine in DMF for (2 × 10 min) at room temperature.

Synthesis of Fmoc-*N*-Me-Leu-Leu-Resin (**R**-**2**). Fmoc-*N*-Me-Leu-OH (1.10 g, 3 mmol), HOAt (0.41 g, 3 mmol), and DIC (0.47 mL, 3 mmol) were dissolved in 15 mL of DMF and reacted at room temperature for 5 min. Following this, the reaction solution was added to the peptide synthesis tube which contained the previously synthesized **R**-**1**, and was placed on a shaker to react for 3 h at room temperature. After filtration, the resin was washed with DCM (3 × 5 mL), DMF (3 × 5 mL), and MeOH (3 × 5 mL). The reaction was monitored using the Kaiser test for the primary amine and the chloranil test for the secondary amine.

Synthesis of Fmoc-Leu-*N*-Me-Leu-Leu-Resin (**R**-**3**). After deprotection of the Fmoc group, the method of synthesizing **R-3** was the same as that of **R-2**. Finally, the Fmoc-Leu-OH was connected to **R**-**2** to synthesize **R**-**3**.

Synthesis of Fmoc-Pro-Leu-*N*-Me-Leu-Leu-Resin (**R**-**4**). After deprotection of the Fmoc group, the method of synthesizing **R-4** was the same as that of **R-2**. Finally, the Fmoc-Pro-OH was connected to **R**-**3** to synthesize **R**-**4**.

Synthesis of Fmoc-Leu-Pro-Leu-*N*-Me-Leu-Leu-Resin (**R**-**5**). After deprotection of the Fmoc group, the method of synthesizing **R-5** was the same as that of R-2. Finally, the Fmoc-Leu-OH was connected to **R**-**4** to synthesize **R**-**5**.

Cleavage of resin peptides. Cleavage was performed using 1% TFA in DCM for (2 × 30 min) at rt. After cleavage, the filtrate was immediately neutralized with 1% pyridine in MeOH and concentrated under reduced pressure. The crude peptide was isolated via precipitation from diethyl ether (20 mL) and collected through centrifuge as a white solid, which can be directly used for a macrocyclization reaction without further purification.

#### 3.2.3. Synthesis of Linear Pentapeptide HO-Leu-Pro-Leu-Pro-Leu-H (**X-2**)

The method of synthesizing **X-2** was the same as that of **X-1**.

#### 3.2.4. Synthesis of Linear Pentapeptide HO-D-Leu-Pro-Leu-Pro-Leu-H (**X-3**)

The method of synthesizing **X-3** was the same as that of **X-1**.

#### 3.2.5. Synthesis of Linear Pentapeptide HO-Leu-Pro-Leu-Pro-D-Leu-H (**X-4**)

The method of synthesizing **X-4** was the same as that of **X-1**.

#### 3.2.6. Synthesis of Linear Pentapeptide HO-Leu-Pro-D-Leu-Pro-Leu-H (**X-5**)

The method of synthesizing **X-5** was the same as that of **X-1**.

#### 3.2.7. Synthesis of Linear Pentapeptide HO-D-Leu-Pro-Leu-Pro-D-Leu-H (**X-6**)

The method of synthesizing **X-6** was the same as that of **X-1**.

#### 3.2.8. Synthesis of Linear Pentapeptide HO-Leu-Pro-D-Leu-Pro-D-Leu-H (**X-7**)

The method of synthesizing **X-7** was the same as that of **X-1**.

#### 3.2.9. Synthesis of Linear Pentapeptide HO-D-Leu-Pro-D-Leu-Pro-D-Leu-H (**X-8**)

The method of synthesizing **X-8** was the same as that of **X-1**.

#### 3.2.10. Synthesis of Cyclo (Leu-N-Me-Leu-Leu-Pro-Leu) (**Z-1**)

Firstly, **X**-**1** (0.10 g, 0.15 mmol) and PyBOP (0.27 g, 0.52 mmol) were dissolved in dichloromethane (400 mL) using a 500-mL round-bottom flask. Subsequently, this mixture reacted at room temperature for 48 h with the addition of DIEA to adjust the pH around 7~8. After that, saturated NaHCO_3_ and H_2_O were used to wash this reaction solution separately. The resultant organic phase was dried with anhydrous Na_2_SO_4_ and concentrated. Then, the obtained solid was purified by RP-HPLC (Agilent Eclipse ZORBAX SB-C18, 5 µm, 9.4 × 250 nm) to obtain 39.4 mg of white powder with a yield of 40.7%. ^1^H NMR (300 MHz, Chloroform-*d*), *δ*_H_ 7.16 (d, *J* = 8.8 Hz, 1H), 6.81 (d, *J* = 8.2 Hz, 1H), 6.67 (d, *J* = 7.6 Hz, 1H), 4.85 (td, *J* = 8.5, 5.1 Hz, 1H), 4.55–4.45 (m, 1H), 4.34 (dd, *J* = 7.2, 3.5 Hz, 1H), 4.25 (td, *J* = 10.0, 4.0 Hz, 1H), 3.76 (dd, *J* = 11.7, 8.9 Hz, 1H), 3.61–3.45 (m, 4H), 3.27 (s, 3H), 2.06–1.37 (m, 14H), 0.85–1.05 (m, 24H); ^13^C NMR (75 MHz, Chloroform-*d*), *δ*_C_ 174.2, 172.6, 172.5, 171.7, 171.0, 77.2, 69.0, 61.2, 54.1, 50.8, 48.8, 47.9, 42.0, 41.5, 39.5, 38.2, 37.7, 32.3, 25.8, 25.1, 25.0, 24.7, 24.4, 23.5, 23.3, 23.2, 23.0, 22.0 (2C), 21.5; HR-ESI-MS *m/z*: 564.4 [M + H]^+^, 580.7 [M + NH_4_]^+^, 586.6 [M + Na]^+^.

#### 3.2.11. Synthesis of Cyclo (Leu-Pro-Leu-Pro-Leu) (**Z-2**)

The method of synthesizing **Z-2** was followed as per that described for **Z-1**. Finally, 34.3 mg of white powder with a yield of 35.5% were obtained. ^1^H NMR (300 MHz, Chloroform-*d*), *δ*_H_ 7.44–7.33 (m, 2H), 7.17 (d, *J* = 8.1 Hz, 1H), 4.80 (ddd, *J* = 22.2, 11.0, 6.4 Hz, 1H), 4.63 (dd, *J* = 11.7, 7.3 Hz, 2H), 4.29 (t, *J* = 9.3 Hz, 1H), 3.72 (dd, *J* = 14.8, 7.8 Hz, 1H), 3.56 (dd, *J* = 10.5, 5.7 Hz, 2H), 2.48–1.37 (m, 19H), 1.13–0.79 (m, 18H); ^13^C NMR (75 MHz, Chloroform-*d*), *δ*_C_ 173.4, 172.6, 171.9, 171.0, 170.8, 61.0, 60.4, 53.2, 49.2, 48.8, 47.7, 47.2, 42.4, 42.3, 40.7, 27.4, 25.2, 25.1, 25.0 (2C), 24.9, 23.5, 23.3 (2C), 22.1, 21.9, 21.3, 21.1; HR-ESI-MS *m/z*: 534.4 [M + H]^+^, 550.7 [M + NH_4_]^+^, 556.8 [M + Na]^+^.

#### 3.2.12. Synthesis of Cyclo (Leu-Pro-Leu-Pro-Leu) (**Z-3**)

The method of synthesizing **Z-3** was followed as per described for **Z**-**1**. Finally, 52.1 mg of white powder with a yield of 53.9% were obtained. ^1^H NMR (300 MHz, Chloroform-*d*), *δ*_H_ 7.62 (d, *J* = 8.7 Hz, 1H), 7.47 (d, *J* = 8.4 Hz, 1H), 6.87 (d, *J* = 9.0 Hz, 1H), 4.80 (t, *J* = 7.7 Hz, 2H), 4.69 (d, *J* = 7.8 Hz, 1H), 4.51 (td, *J* = 10.0, 4.1 Hz, 1H), 4.25 (t, *J* = 6.6 Hz, 1H), 3.89–3.51 (m, 4H), 2.39 (t, *J* = 8.8 Hz, 1H), 2.29–1.82 (m, 8H), 1.78–1.36 (m, 8H), 0.93 (d, *J* = 6.6 Hz, 18H); ^13^C NMR (75 MHz, Chloroform-*d*), *δ*_C_ 172.6, 172.0, 171.9, 171.9, 171.0, 61.0, 59.9, 51.5, 49.3, 49.1, 47.6, 47.4, 41.0, 40.9, 40.4, 29.2, 27.0, 25.3, 25.1, 25.0, 24.7, 24.6, 23.5, 23.1, 23.0, 22.4, 21.7, 21.4; HR-ESI-MS *m/z*: 534.7 [M + H]^+^, 550.7 [M + NH_4_]^+^, 556.8 [M + Na]^+^.

#### 3.2.13. Synthesis of Cyclo (Leu-Pro-D-Leu-Pro-Leu) (**Z-4**)

The method of synthesizing **Z-4** was followed as per that described for **Z-1**. Finally, 35.2 mg of white powder with a yield of 36.4% were obtained. ^1^H NMR (300 MHz, Chloroform-*d*), *δ*_H_ 8.07 (d, *J* = 8.0 Hz, 1H), 7.16 (d, *J* = 9.1 Hz, 1H), 6.57 (d, *J* = 8.8 Hz, 1H), 4.86 (d, *J* = 7.6 Hz, 1H), 4.79 (t, *J* = 7.9 Hz, 1H), 4.72–4.60 (m, 1H), 4.51 (td, *J* = 9.1, 5.0 Hz, 1H), 4.37 (dd, *J* = 8.5, 3.9 Hz, 1H), 3.98 (dt, *J* = 11.1, 6.0 Hz, 1H), 3.61 (td, *J* = 9.3, 4.1 Hz, 1H), 3.56–3.42 (m, 2H), 2.29 (qt, *J* = 9.2, 4.6 Hz, 2H), 2.16 (dt, *J* = 15.5, 8.0 Hz, 1H), 2.10–1.84 (m, 6H), 1.81 -1.43 (m, 7H), 1.32 (dt, *J* = 12.0, 6.7 Hz, 1H), 1.05–0.83 (m, 18H); ^13^C NMR (75 MHz, Chloroform-*d*), *δ*_C_ 172.9, 172.2, 171.4, 171.3, 171.0, 77.3, 62.0, 58.7, 51.5, 50.3, 49.5, 47.3, 40.2, 40.0, 39.7, 29.7, 25.8, 25.2, 24.9 (2C), 24.7 (2C), 23.3, 23.2, 22.8, 22.5, 21.6, 21.5; HR-ESI-MS *m/z*: 534.4 [M + H]^+^, 550.7 [M + NH_4_]^+^, 556.8 [M + Na]^+^.

#### 3.2.14. Synthesis of Cyclo (Leu-Pro-Leu-Pro-D-Leu) (**Z-5**)

The method of synthesizing **Z-5** was followed as per that described for **Z-1**. Finally, 57.0 mg of white powder with a yield of 58.9% were obtained. ^1^H NMR (300 MHz, Chloroform-*d*), *δ*_H_ 8.62 (d, *J* = 9.0 Hz, 1H), 7.12 (d, *J* = 8.7 Hz, 1H), 6.93 (d, *J* = 6.7 Hz, 1H), 4.77 (q, *J* = 7.9 Hz, 1H), 4.66–4.50 (m, 3H), 4.40 (dt, *J* = 8.6, 6.1 Hz, 1H), 4.28–4.02 (m, 2H), 3.68 (td, *J* = 9.2, 3.9 Hz, 1H), 3.47 (td, *J* = 9.5, 6.7 Hz, 1H), 2.41–2.17 (m, 1H), 2.17–1.81 (m, 8H), 1.81–1.50 (m, 6H), 1.51–1.35 (m, 2H), 1.02–0.84 (m, 18H); ^13^C NMR (75 MHz, Chloroform-*d*), *δ*_C_ 174.0, 172.7, 171.7, 171.0, 170.9, 60.6, 59.2, 50.8, 49.9, 48.9, 47.5, 46.8, 41.3, 39.5, 37.8, 29.9, 28.1, 25.1, 24.8 (3C), 24.0, 23.3, 23.0 (2C), 22.5, 22.4, 22.0; HR-ESI-MS *m/z*: 534.4 [M + H]^+^, 550.7 [M + NH_4_]^+^, 556.8 [M + Na]^+^.

#### 3.2.15. Synthesis of Cyclo (D-Leu-Pro-Leu-Pro-D-Leu) (**Z-6**)

The method of synthesizing **Z-6** was followed as per that described for **Z-1**. Finally, 33.4 mg of white powder with a yield of 34.5% were obtained. ^1^H NMR (300 MHz, Chloroform-*d*), *δ*_C_ 8.00 (d, *J* = 7.3 Hz, 1H), 7.59 (d, *J* = 6.8 Hz, 1H), 7.27 (d, *J* = 9.2 Hz, 1H), 4.77 (td, *J* = 9.5, 4.2 Hz, 1H), 4.64 (d, *J* = 7.6 Hz, 1H), 4.55 (d, *J* = 7.5 Hz, 2H), 4.24–4.12 (m, 1H), 4.07 (dt, *J* = 11.5, 6.3 Hz, 1H), 3.91 (q, *J* = 8.5 Hz, 1H), 3.63–3.41 (m, 2H), 2.48–1.29 (m, 18H), 1.11–0.76 (m, 18H); ^13^C NMR (75 MHz, Chloroform-*d*), *δ*_C_ 174.4, 172.4, 172.1, 171.8, 171.3, 60.8, 59.8, 55.8, 50.2, 49.1, 47.4, 47.0, 41.0, 39.8, 39.1, 29.7, 26.7, 25.2, 25.1, 24.9, 24.8, 24.3, 23.3, 23.2, 23.0, 22.0, 21.7 (2C); HR-ESI-MS *m/z*: 534.4 [M + H]^+^, 550.7 [M + NH_4_]^+^, 556.8 [M + Na]^+^.

#### 3.2.16. Synthesis of Cyclo (Leu-Pro-D-Leu-Pro-D-Leu) (**Z-7**)

The method of synthesizing **Z-7** was followed as per that described for **Z-1**. Finally, 52.8 mg of white powder with a yield of 54.6% were obtained. ^1^H NMR (300 MHz, Chloroform-*d*), *δ*_H_ 8.03 (d, *J* = 8.3 Hz, 1H), 7.17 (d, *J* = 9.0 Hz, 1H), 6.60 (d, *J* = 8.8 Hz, 1H), 4.79–4.86 (m, 1H), 4.74 (d, *J* = 7.9 Hz, 1H), 4.69–4.60 (m, 1H), 4.46 (td, *J* = 9.3, 4.7 Hz, 1H), 4.31 (dd, *J* = 9.0, 3.9 Hz, 1H), 3.97 (dd, *J* = 10.1, 5.4 Hz, 1H), 3.40–3.62 (m, 3H), 2.39–2.18 (m, 2H), 2.18–2.08 (m, 1H), 2.08–1.79 (m, 6H), 1.75–1.42 (m, 7H), 1.29 (ddd, *J* = 13.6, 9.7, 3.8 Hz, 1H), 1.03–0.81 (m, 18H); ^13^C NMR (75 MHz, Chloroform-*d*), *δ*_C_ 172.6, 172.3, 171.4, 171.3, 171.2, 62.0, 58.6, 51.5, 50.1, 49.5, 47.2, 46.4, 40.3, 40.0, 39.7, 29.6, 25.6, 25.2, 25.0, 24.9, 24.7, 24.7, 23.3, 23.2, 22.8, 22.5, 21.6, 21.5; HR-ESI-MS *m/z*: 534.4 [M + H]^+^, 550.7 [M + NH_4_]^+^, 556.8 [M + Na]^+^.

#### 3.2.17. Synthesis of Cyclo (D-Leu-Pro-D-Leu-Pro-D-Leu) (**Z-8**)

The method of synthesizing **Z-8** was followed as per that described for **Z-1**. Finally, 44.7 mg of white powder with a yield of 46.2% were obtained. ^1^H NMR (300 MHz, Chloroform-*d*), *δ*_H_ 7.94 (d, *J* = 8.4 Hz, 1H), 7.17 (d, *J* = 9.1 Hz, 1H), 6.53 (d, *J* = 8.8 Hz, 1H), 4.85 (d, *J* = 7.5 Hz, 1H), 4.78 (d, *J* = 8.1 Hz, 1H), 4.68 (d, *J* = 8.3 Hz, 1H), 4.50 (td, *J* = 9.4, 4.6 Hz, 1H), 4.34 (dd, *J* = 8.8, 4.0 Hz, 1H), 3.97 (dt, *J* = 11.9, 6.2 Hz, 1H), 3.60–3.43 (m, 3H), 2.31 (td, *J* = 12.9, 5.2 Hz, 2H), 2.16 (dt, *J* = 11.8, 8.1 Hz, 1H), 2.11–1.82 (m, 6H), 1.80–1.42 (m, 7H), 1.32 (ddd, *J* = 13.6, 9.8, 3.8 Hz, 1H), 1.25–0.85 (m, 18H); ^13^C NMR (75 MHz, Chloroform-*d*), *δ*_C_ 172.7, 172.1, 171.4, 171.3, 171.1, 62.0, 58.5, 51.4, 50.1, 49.5, 47.3, 46.5, 40.4, 40.0, 39.8, 29.7, 25.4, 25.2, 25.0, 24.9, 24.7, 24.7, 23.3, 23.2, 22.8, 22.5, 21.5 (2C); HR-ESI-MS *m/z*: 534.4 [M + H]^+^, 550.7 [M + NH_4_]^+^, 556.8 [M + Na]^+^.

### 3.3. In Vitro Cytotoxic Assays

Cell culture: MCF-7/MD-MAB-231 (human breast cancer cell line ATCC HTB-22/HTB-26), Hep G2 (human liver cancer cell line ATCC HB-8065), Hela (human cervical carcinoma cell line ATCC CCL-2), A459 (human lung cancer cell line ATCC CCL-185), and HUVEC (primary umbilical vein endothelial cell line ATCC PCS-100-010) were obtained from the Jinan Department of Pathology, University School of Medicine (Jinan University, Guangzhou, China). The cells were cultured in a Modified Eagle’s Medium (DMEM) including 10% fetal bovine serum, 1% penicillin-streptomycin, and antifungal agent, and cultured under standard conditions (5% CO_2_ atmosphere and temperature at 37 °C).

### 3.4. MTT Assay

The MTT method was used to evaluate the cytotoxicities of galaxamide and its analogues on the five cancerous cells. In this experiment, the number of regulated cells was 2 × 10^3^ cells/well. Then, the cells, as well as galaxamide and its analogues of different concentrations (0, 2.5, 5, and 10 μg/mL), were seeded on a 96-well cell culture plate and cultured in a 37 °C, 5% CO_2_ incubator for 72 h. After that, 20 µL/well of MTT solution (5 mg/mL phosphate-buffered saline) was added and incubated for 5 h. The medium was discarded and replaced with 100 µL/well of DMSO to dissolve the formazan salt. The color intensity of the cell culture at 570 nm was measured using a microplate spectrophotometer (SpectroAma™ 250, Winooski, VT, USA).

### 3.5. Flow Cytometry Experiments

The concentration of 1 × 10^4^ cells per well of MCF-7 cells were seeded in 96-well plates. The cell cultures were added to the compounds with a concentration of 0, 2.5, 5, and 10 µg/mL, and incubated for 72 h. After that, 5 µL of Annexin V-fluorescein isothiocyanate (FITC) and 5 µL of PI were added for dual staining of the cells for analysis by FACS flow cytometry. Cell cycles and apoptotic cells were determined by counting the FITC-positive and PI-negative fractions.

### 3.6. Cell Apoptotic Analysis

The apoptotic cells were quantified according to the instructions provided in an Annexin V-FITC (Sigma-Aldrich, St. Louis, MO, USA) cell apoptosis assay kit. Briefly, about 1.5 × 10^5^ cells were plated in 96-well plates and treated with galaxamide and its representative analogues (0, 2.5, 5, and 10 µg/mL) for 48 h. The cells were then resuspended in a 200-mL binding buffer. Afterward, 5 mL of annexin V-fluorescein isothiocyanate (FITC) was added, followed by incubation in darkness at room temperature for 10 min. The cells were again resuspended in 200 mL binding buffer and stained with 5 mL PI. The prepared cells were then analyzed using a flow cytometry (Coulter Epics Elite, Miami, FL, USA). The cells in the FITC-positive and PI-negative fractions were regarded as apoptotic cells.

### 3.7. Hoechst 33,342 Staining

Test compounds at 0, 2.5, 5, and 10 µg/mL were added to the cell cultures which included the exponentially growing MCF-7 cells (1 × 10^5^), and incubated for 48 h. After that, the cell culture was washed with 1 mL of PBS and then added with 3 mL of 4% paraformaldehyde solution for 15 min. Then, 1 mL of the prepared Hoechst 33,342 staining solution was added. The resulting mixture was incubated at room temperature for 15 min in the dark. Ultimately, cell staining was observed with a fluorescence microscope.

## 4. Conclusions

In this paper, eight new cyclic pentapeptides (**Z-1**~**Z-8**) were synthesized, and were designed by replacing one or two *N*-Me-*L*-leucine with *L*-proline or changing the stereo configuration of leucine. The anticancer activities of these new compounds were also tested against five human cancer cell lines. The results showed that the inhibitory effects of most analogues were much better than that of galaxamide. Moreover, all tested compounds exhibited low cytotoxic activity against the normal liver cell line HUVEC. Compound **Z-6** displayed the strongest inhibitory effects against HepG2, MCF-7, and MD-MAB-231 cells with IC_50_ values of 2.91 ± 0.17, 1.65 ± 0.30, 4.59 ± 0.27 μg/mL, respectively. These galaxamide analogues arrested the cell cycle in the G0/G1 phase and induced apoptosis, which was later confirmed by the representative compounds **Z-3** and **Z-6**. In our preliminary work, a series of galaxamide analogues had been synthesized by replacing one of the amino acids to phenylalanine, the cytotoxic activities of which were also tested against several cancerous cell lines. [14,16] The results showed that most of these newly synthesized compounds exhibited stronger inhibitory activities against MCF-7 cells than the previous ones, which suggests that proline might be the active group of these analogues against MCF-7 cells. Th structure-activity relationship needs further investigation.

## Figures and Tables

**Table 1:** The inhibitory effects of galaxamide and its analogues against several cell lines.

Compd.	IC_50_ (μg/mL)					
	MCF-7	HepG2	MD-MBA-231	Hela	A549	HUVEC
**galaxamide**	11.33 ± 2.95	5.20 ± 0.52	8.73 ± 0.29	8.53 ± 0.73	6.99 ± 0.63	>40
**Z**-**1**	5.85 ± 1.28	7.57 ± 0.17	17.81 ± 0.60	11.56 ± 0.65	4.92 ± 0.84	>40
**Z**-**2**	4.68 ± 1.22	11.95 ± 0.64	14.45 ± 1.10	14.50 ± 0.36	7.97 ± 2.06	>40
**Z**-**3**	2.25 ± 0.42	5.05 ± 0.45	6.34 ± 0.60	5.57 ± 0.45	6.43 ± 0.43	>40
**Z**-**4**	4.88 ± 0.58	15.23 ± 1.14	22.09 ± 1.12	7.38 ± 0.83	9.85 ± 0.95	>40
**Z**-**5**	13.43 ± 1.96	14.98 ± 0.93	8.48 ± 1.12	12.12 ± 0.95	9.50 ± 1.13	>40
**Z**-**6**	1.65 ± 0.30	2.91 ± 0.17	4.59 ± 0.27	5.69 ± 0.37	5.96 ± 0.41	38.72 ± 0.67
**Z**-**7**	13.28 ± 3.28	13.85 ± 1.99	16.52 ± 1.70	18.21 ± 1.35	9.97 ± 1.00	>40
**Z**-**8**	7.82 ± 1.18	9.00 ± 0.83	20.16 ± 2.24	7.97 ± 0.61	15.88 ± 1.42	>40

## Data Availability

Data are contained within the article or Supplementary Material.

## Supplementary Materials

The following supporting information can be downloaded at: www.mdpi.com/article/10.3390/md20030158/s1, Figures S1–S24: The analyzed data of HR-ESI-MS and NMR spectra of compounds **Z****-1**~**Z****-8**.
