# Supplementary material for: Synthesis of Marine Cyclopeptide Galaxamide Analogues as Potential Anticancer Agents
Source: Mar Drugs. 2022 Feb 22;20(3):158. doi: 10.3390/md20030158 (PMC8949366; doi:10.3390/md20030158)
Supplement: Supplementary file 1 [file marinedrugs-20-00158-s001.zip › marinedrugs-1584115-supplementary.pdf]

# Supplementary Material

# Synthesis of Marine Cyclopeptide Galaxamide Analogues as Potential Anticancer Agents

Daichun Li,[#] Xiaojian Liao,[#] Shenghui Zhong, Bingxin Zhao[*] and Shihai Xu[*]

*Department of Chemistry, College of Chemistry and Materials Science, Jinan University, Guangzhou 510632, China*

** Corresponding authors. E-mail addresses: zbx840622@163.com; txush@jnu.edu.cn; zh.86.jiang@gmail.com.*

[#] These authors contributed equally to this work.

# Contents

**Figure S1**. HR-ESI-MS spectrum of **Z-1**……………………………………………….3
**Figure S2**. $^{1}$H NMR spectrum of **Z-1**...............................................................3
**Figure S3**. $^{13}$C NMR spectrum of **Z-1**.............................................................4
**Figure S4**. HR-ESI-MS spectrum of **Z-2**.......................................................4
**Figure S5**. $^{1}$H NMR spectrum of **Z-2**...............................................................5
**Figure S6**. $^{13}$C NMR spectrum of **Z-2**.............................................................5
**Figure S7**. HR-ESI-MS spectrum of **Z-3**……………………………………………….6
**Figure S8**. $^{1}$H NMR spectrum of **Z-3**...............................................................6
**Figure S9**. $^{13}$C NMR spectrum of **Z-3**.............................................................7
**Figure S10**. HR-ESI-MS spectrum of **Z-4**………………………………………..…..…7
**Figure S11**. $^{1}$H NMR spectrum of **Z-4**.............................................................8
**Figure S12**. $^{13}$C NMR spectrum of **Z-4**...........................................................8
**Figure S13**. HR-ESI-MS spectrum of **Z-5**……………………………………….…..…9
**Figure S14**. $^{1}$H NMR spectrum of **Z-5**.............................................................9
**Figure S15**. $^{13}$C NMR spectrum of **Z-5**...........................................................10
**Figure S16**. HR-ESI-MS spectrum of **Z-6**…………………………………….......10
**Figure S17**. $^{1}$H NMR spectrum of **Z-6**.............................................................11
**Figure S18**. $^{13}$C NMR spectrum of **Z-6**...........................................................11
**Figure S19**. HR-ESI-MS spectrum of **Z-7**……………………………………..……...12
**Figure S20**. $^{1}$H spectrum of **Z-7**.....................................................................12
**Figure S21**. $^{13}$C NMR spectrum **Z-7**...............................................................13
**Figure S22**. HR-ESI-MS spectrum of **Z-8**……………………………………..….......13
**Figure S23**. $^{1}$H spectrum of **Z-8**.....................................................................14
**Figure S24**. $^{13}$C NMR spectrum **Z-8**...............................................................14

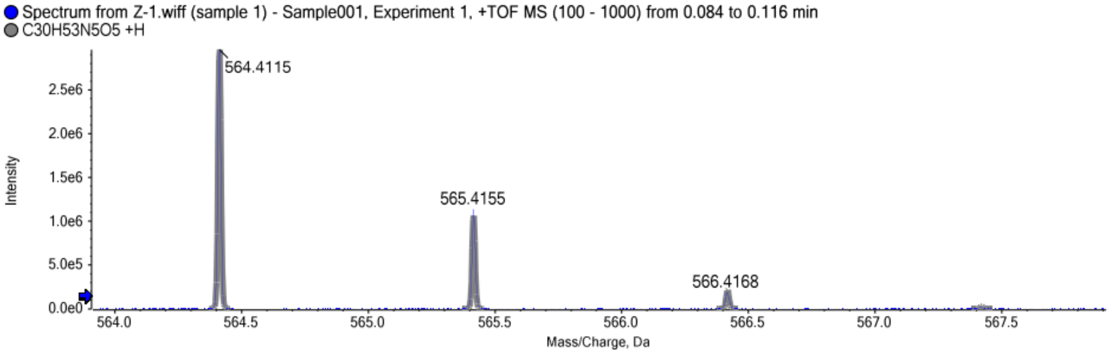


**Figure S1**. HR-ESI-MS spectrum of **Z-1**

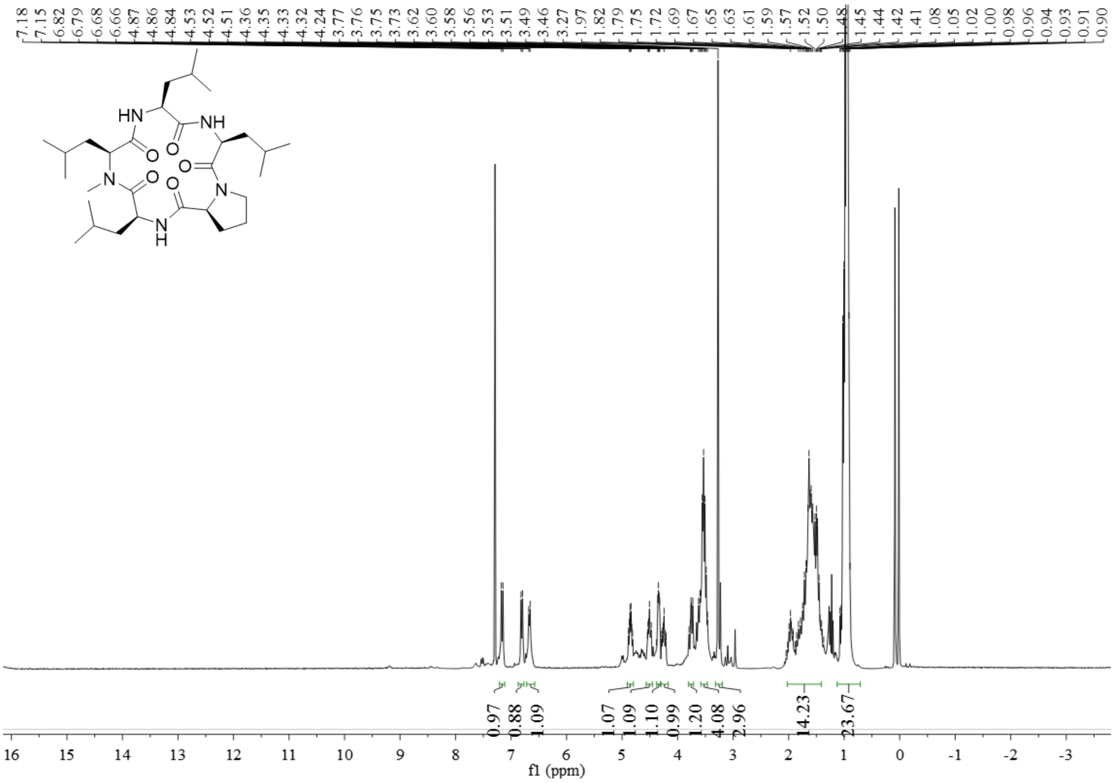


**Figure S2**. $^{1}$H NMR spectrum of **Z-1**

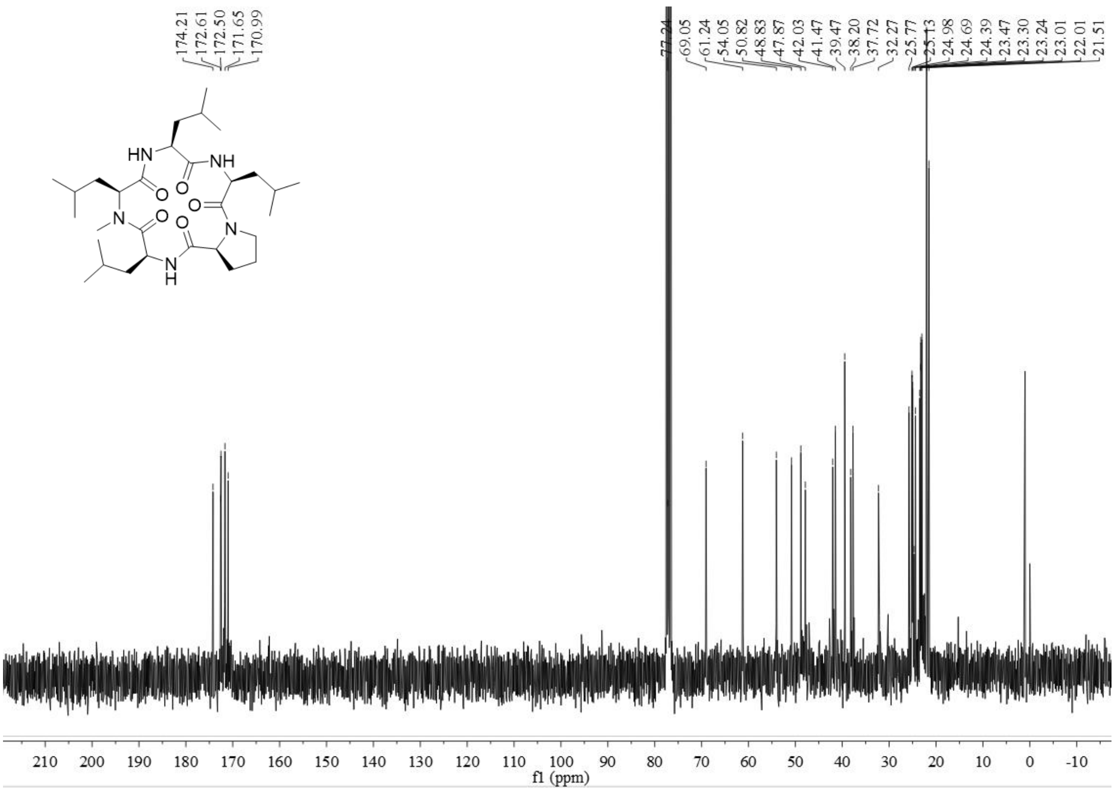


**Figure S3**. $^{13}$C NMR spectrum of **Z-1**

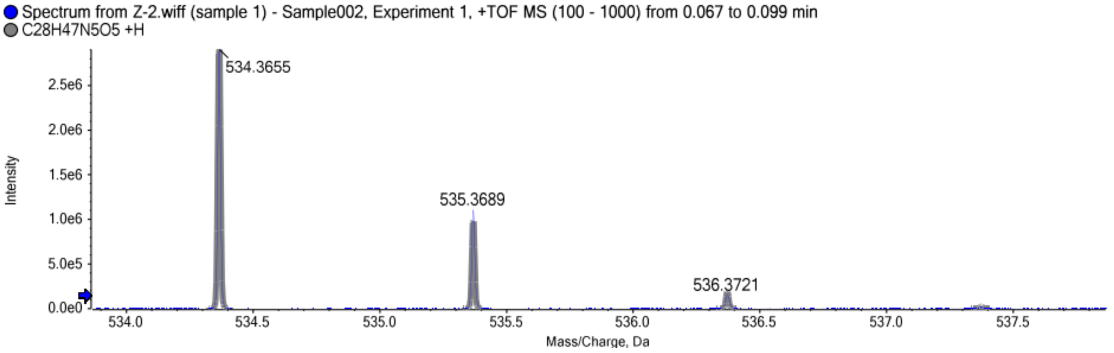


**Figure S4**. HR-ESI-MS spectrum of **Z-2**

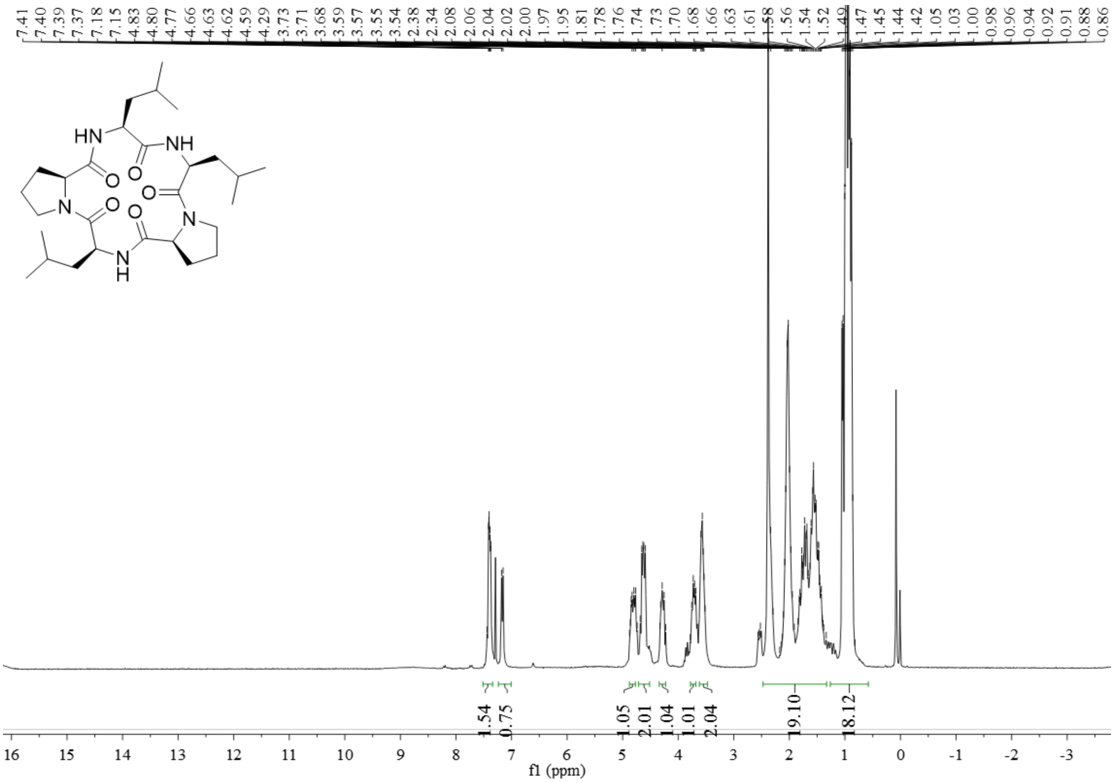


**Figure S5**. $^{1}$H NMR spectrum of **Z-2**

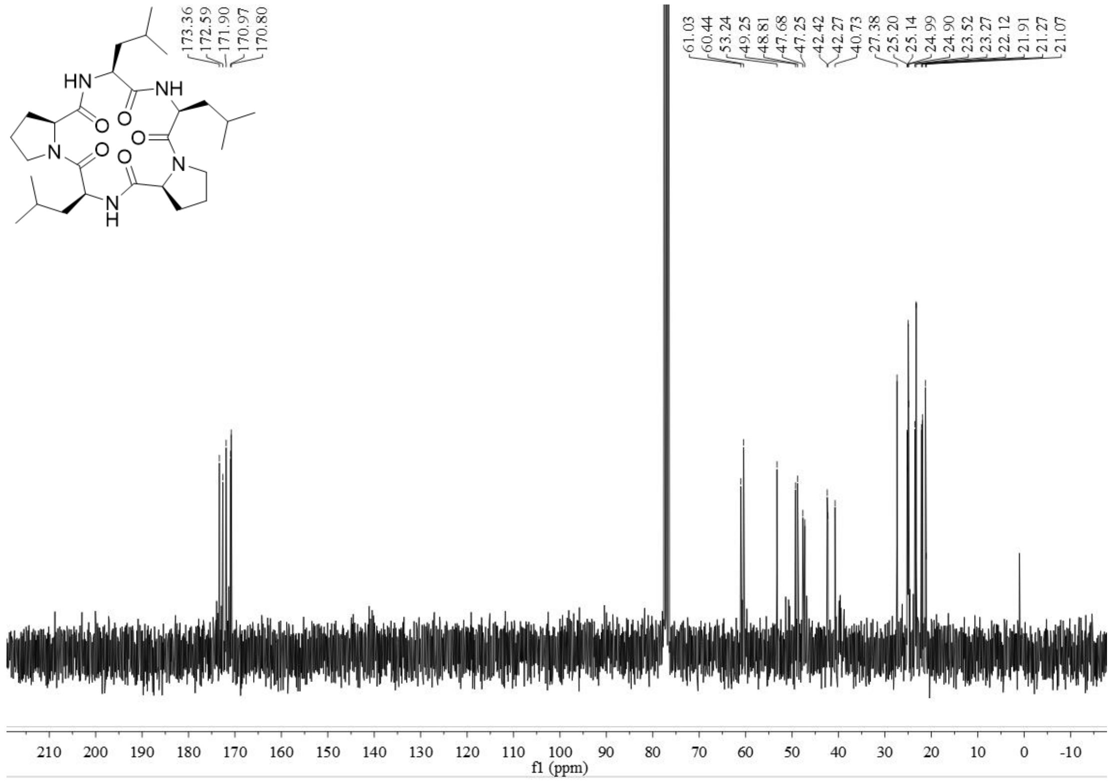


**Figure S6**. $^{13}$C NMR spectrum of **Z-2**

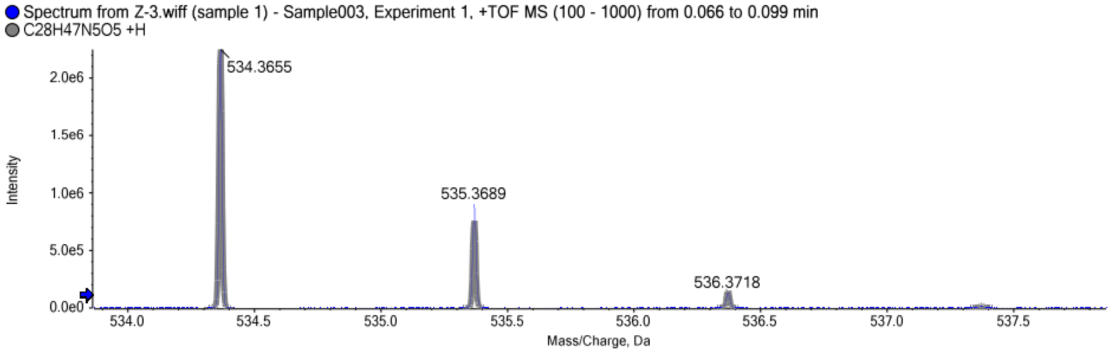


**Figure S7**. HR-ESI-MS spectrum of **Z-3**

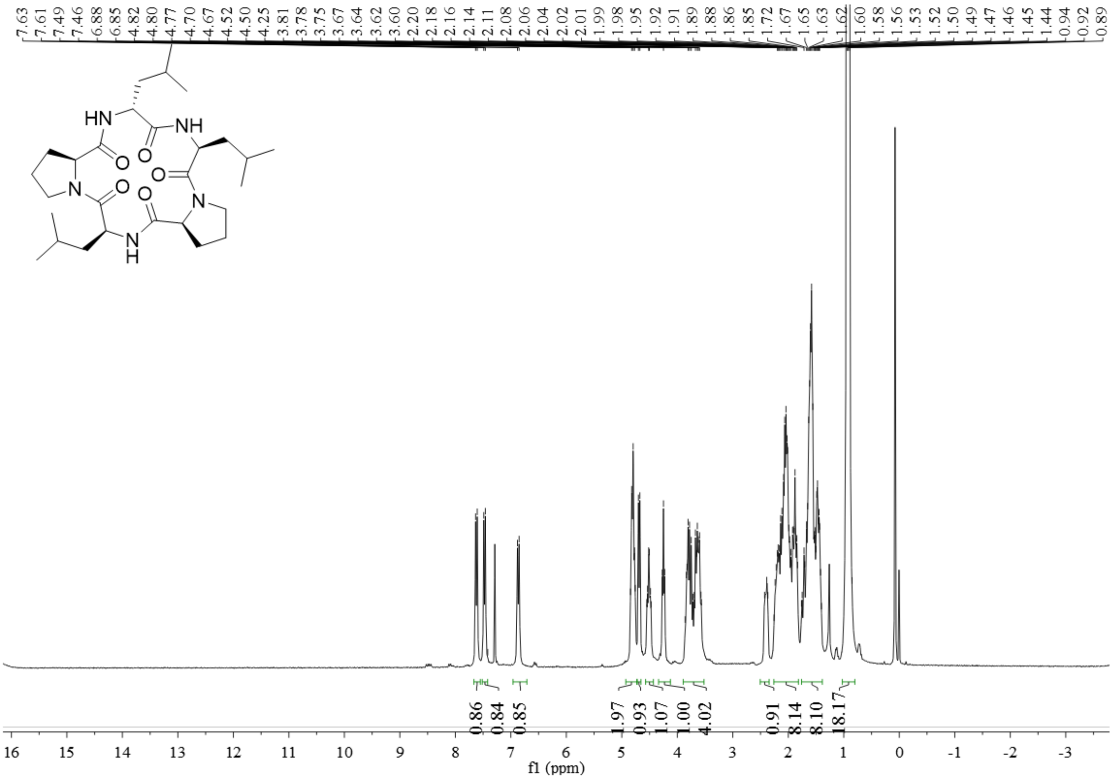


**Figure S8**. $^{1}$H NMR spectrum of **Z-3**

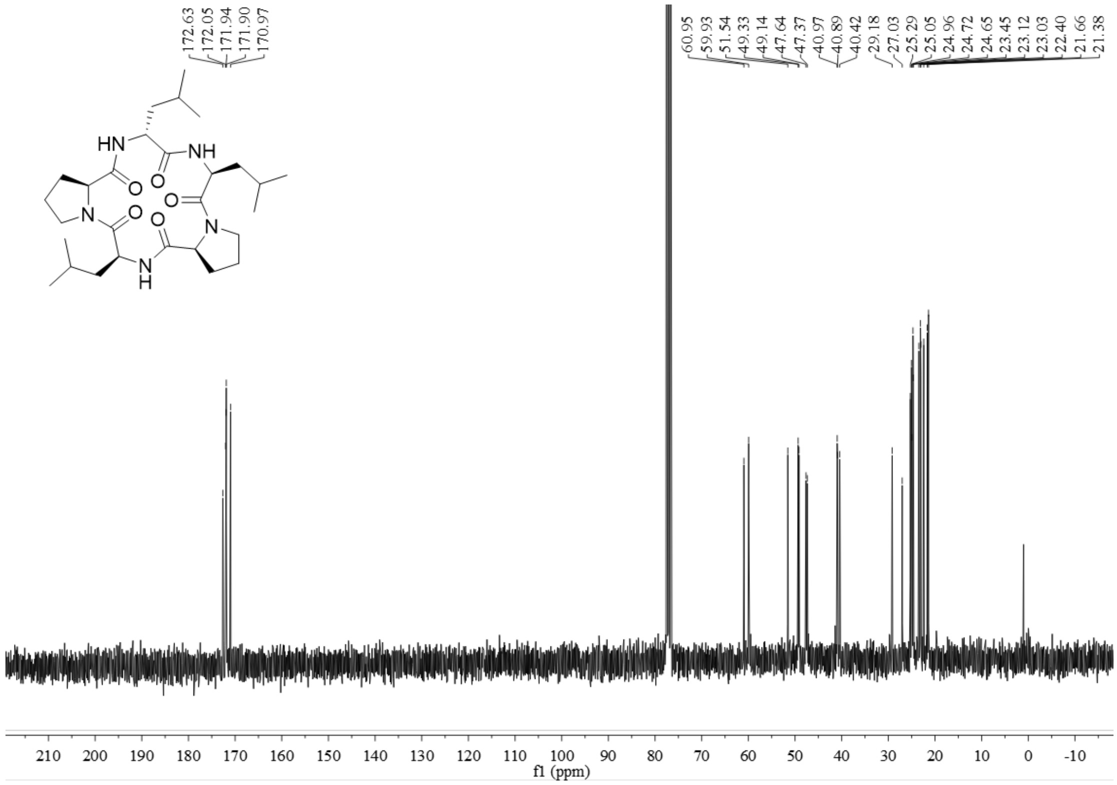


**Figure S9**. $^{13}$C NMR spectrum of **Z-3**

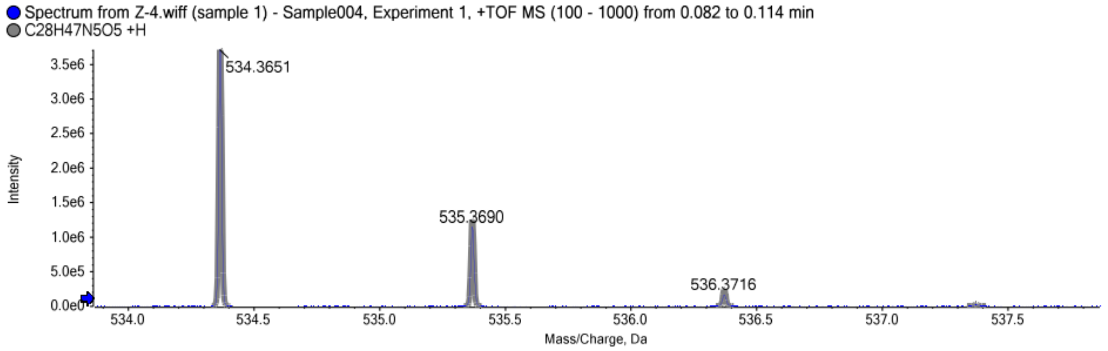


**Figure S10**. HR-ESI-MS spectrum of **Z-4**

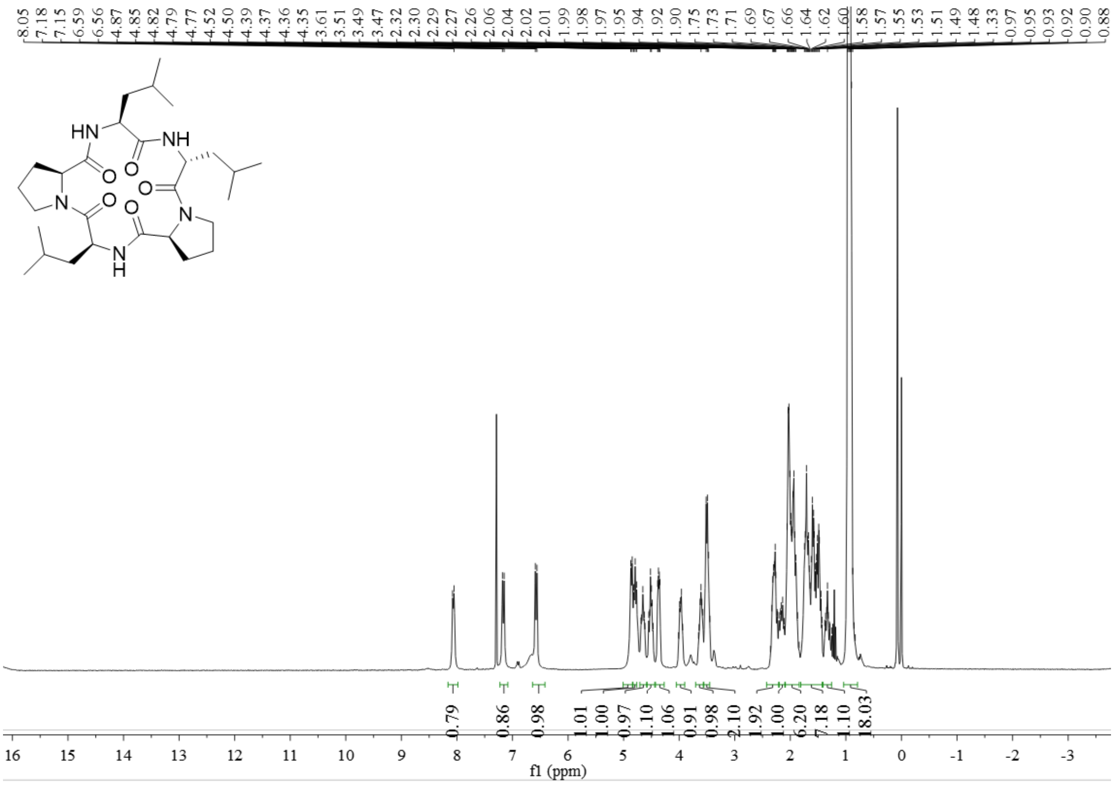

**Figure S11**. $^{1}$H NMR spectrum of **Z-4**

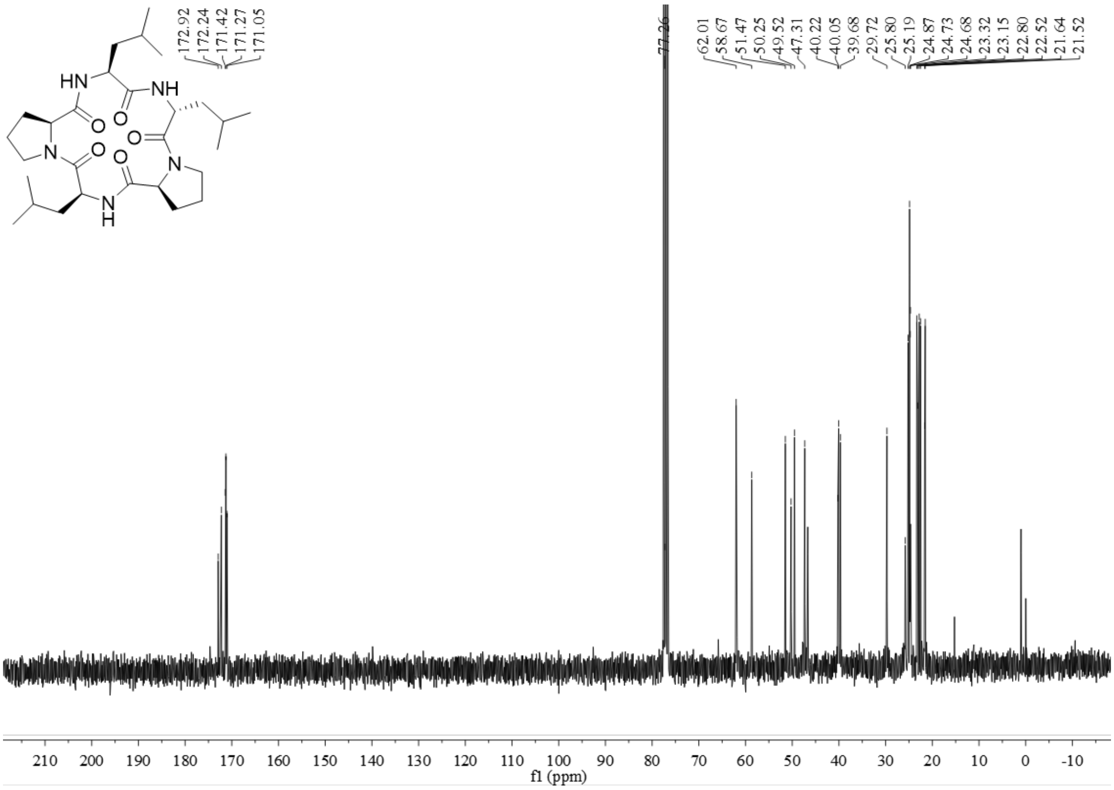

**Figure S12**. $^{13}$C NMR spectrum of **Z-4**

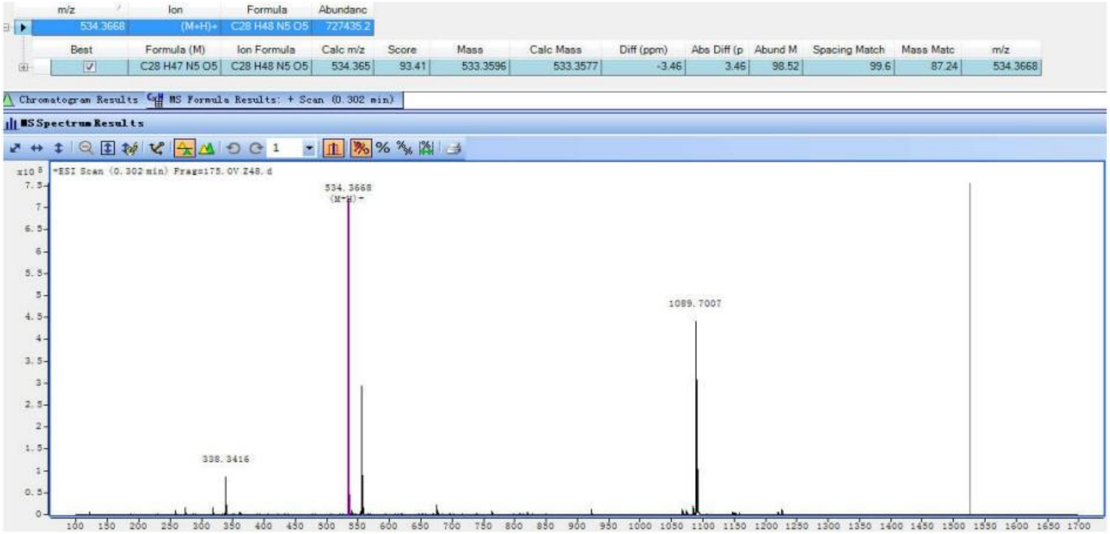


**Figure S13**. HR-ESI-MS spectrum of **Z-5**

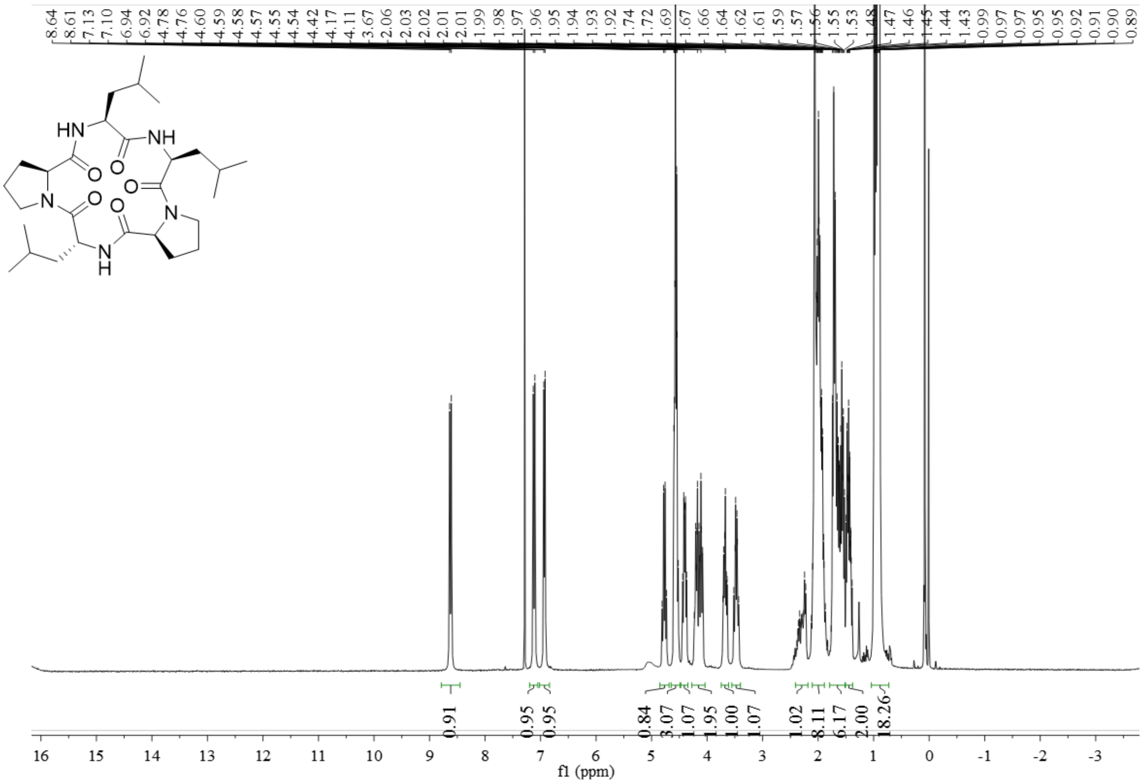


**Figure S14**. $^{1}$H NMR spectrum of **Z-5**

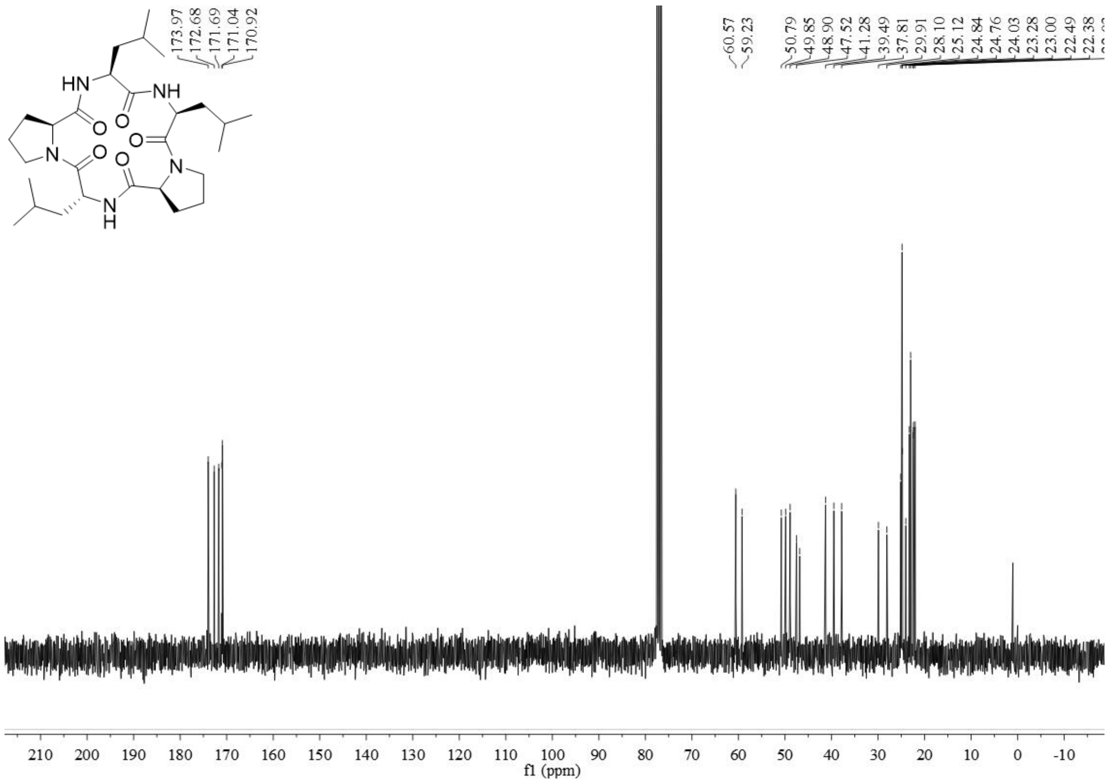


**Figure S15**. $^{13}$C NMR spectrum of **Z-5**

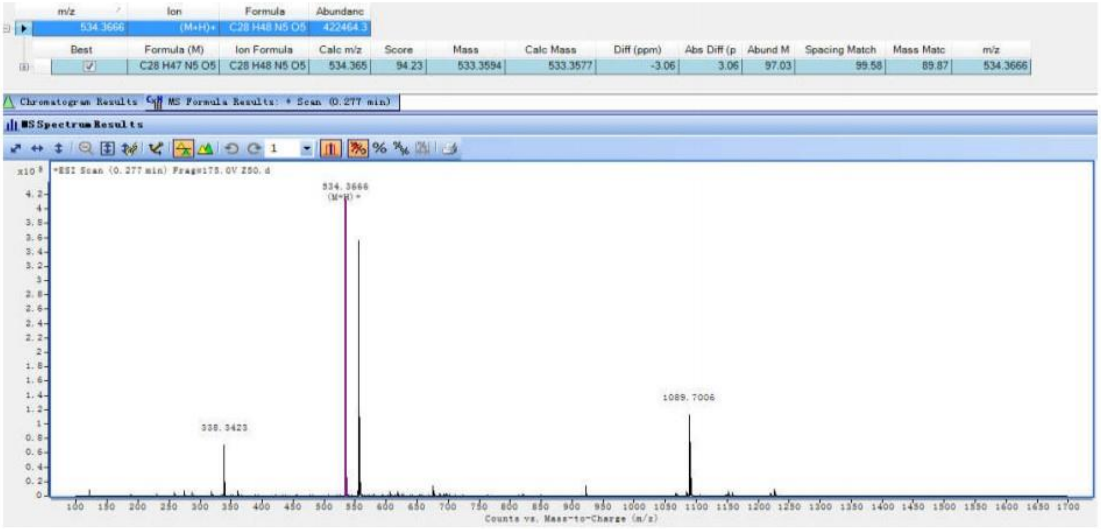


**Figure S16**. HR-ESI-MS spectrum of **Z-6**

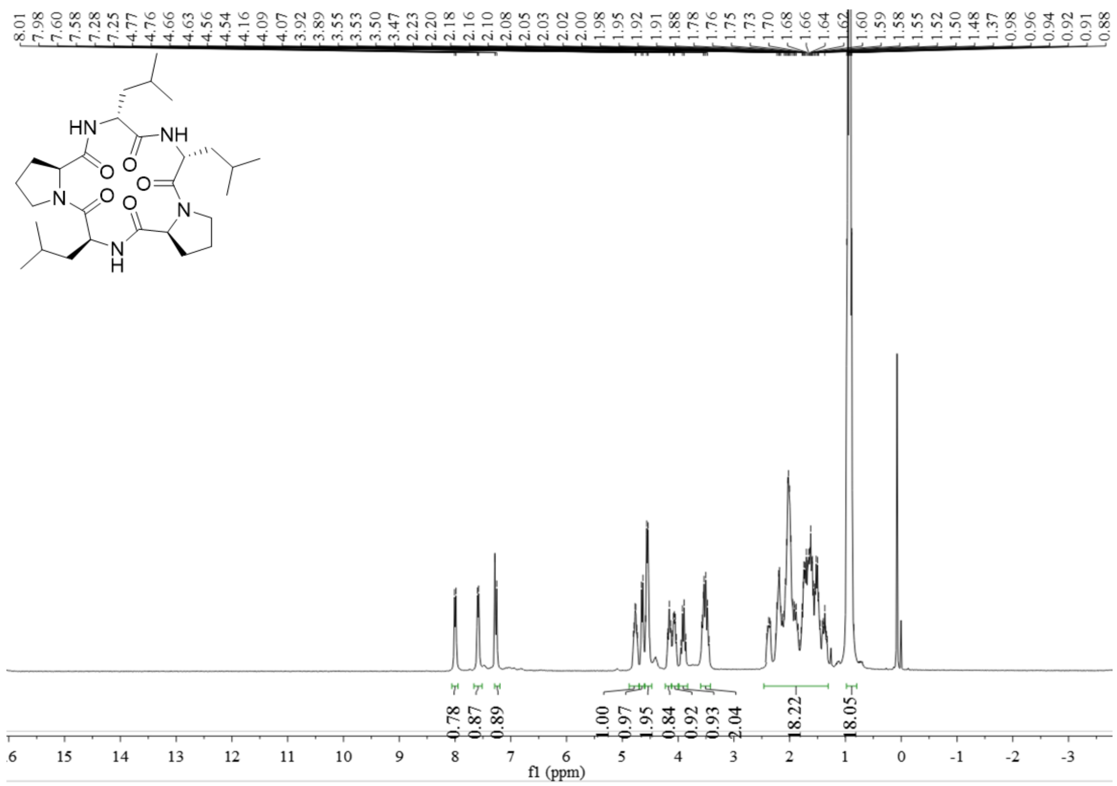


**Figure S17**. $^{1}$H NMR spectrum of **Z-6**

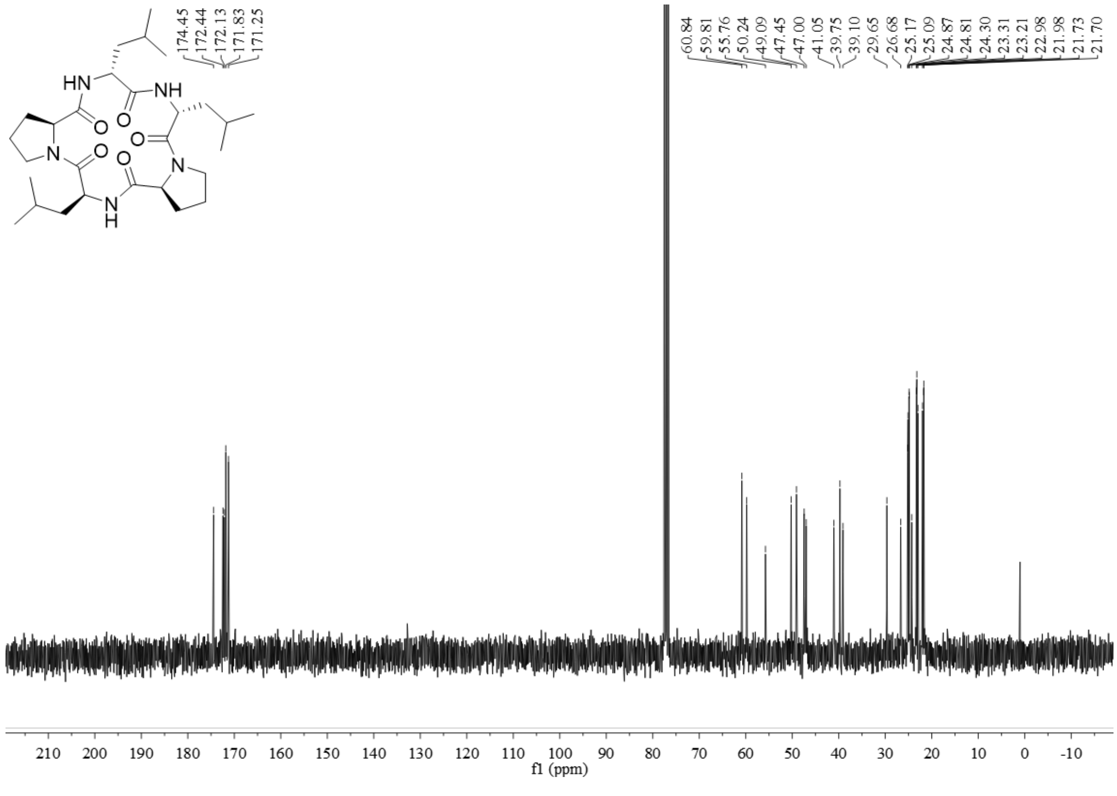


**Figure S18**. $^{13}$C NMR spectrum of **Z-6**

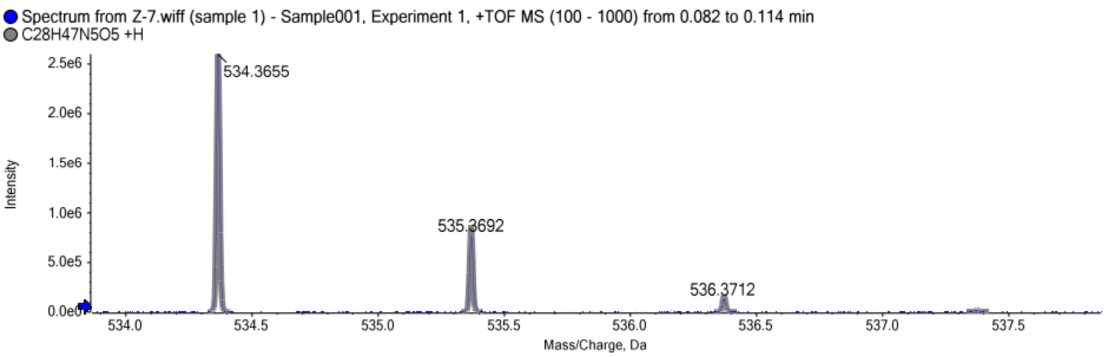


**Figure S19**. HR-ESI-MS spectrum of **Z-7**

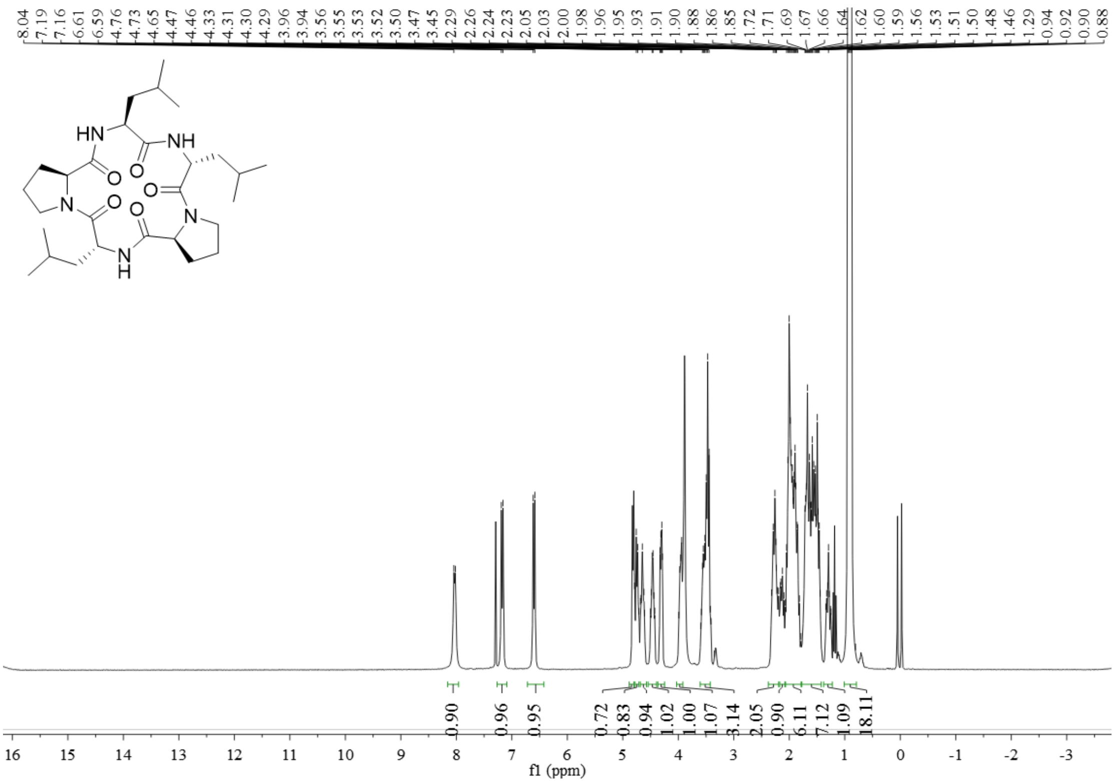


**Figure S20**. $^1$H NMR spectrum of **Z-7**

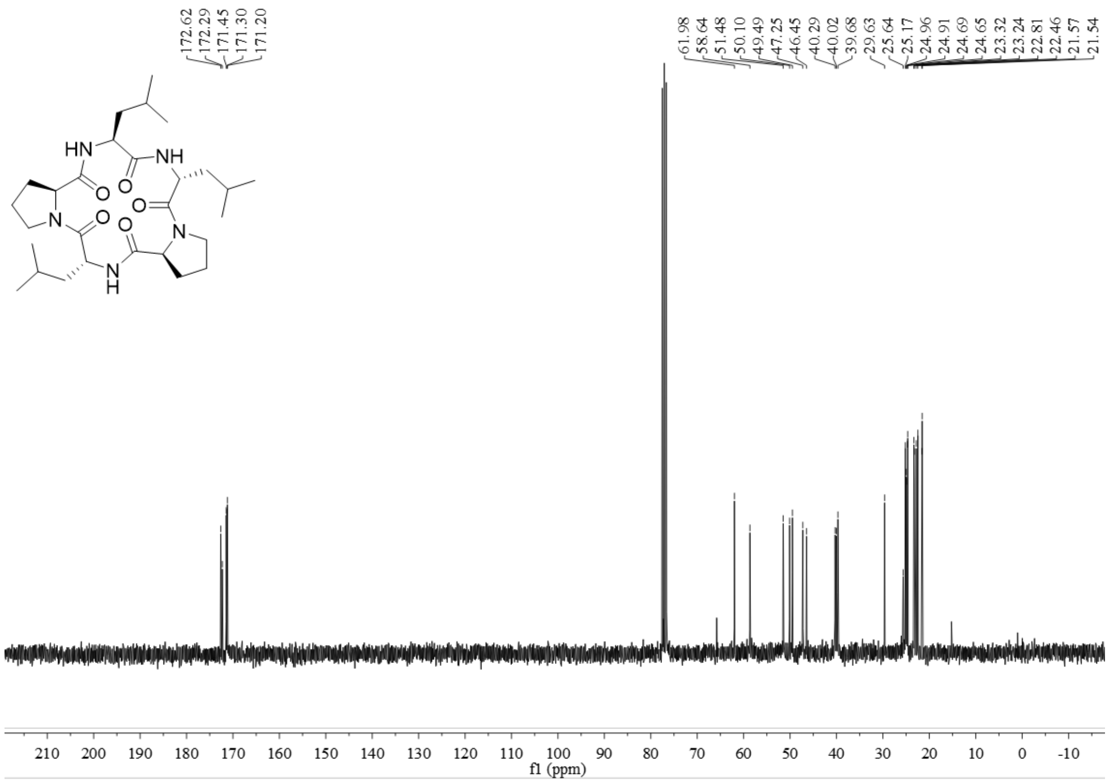


**Figure S21**. $^{13}$C NMR spectrum of **Z-7**

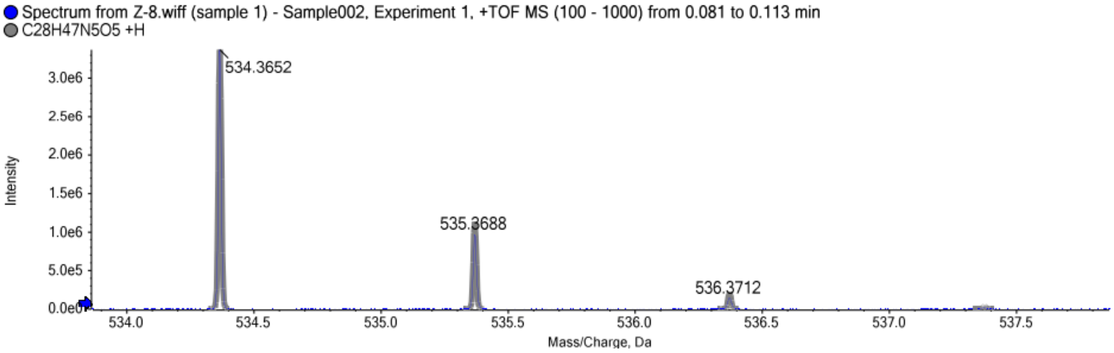


**Figure S22**. HR-ESI-MS spectrum of **Z-8**

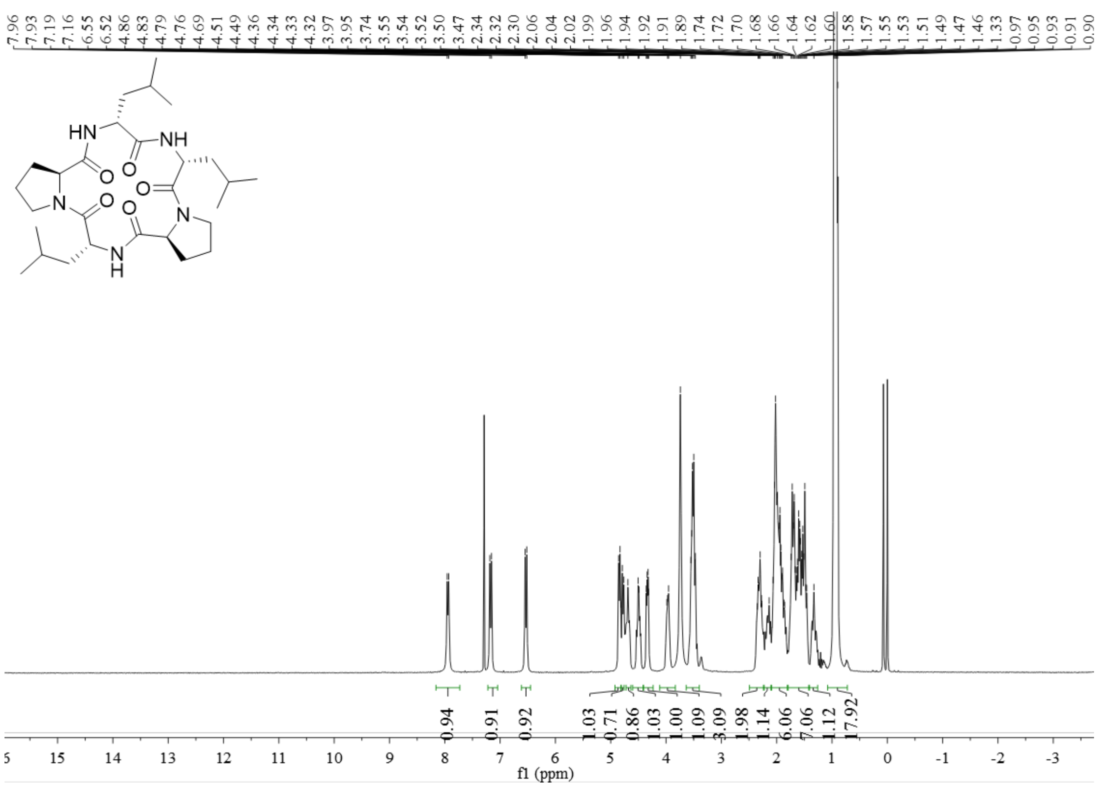


**Figure S23**. $^{1}$H NMR spectrum of **Z-8**

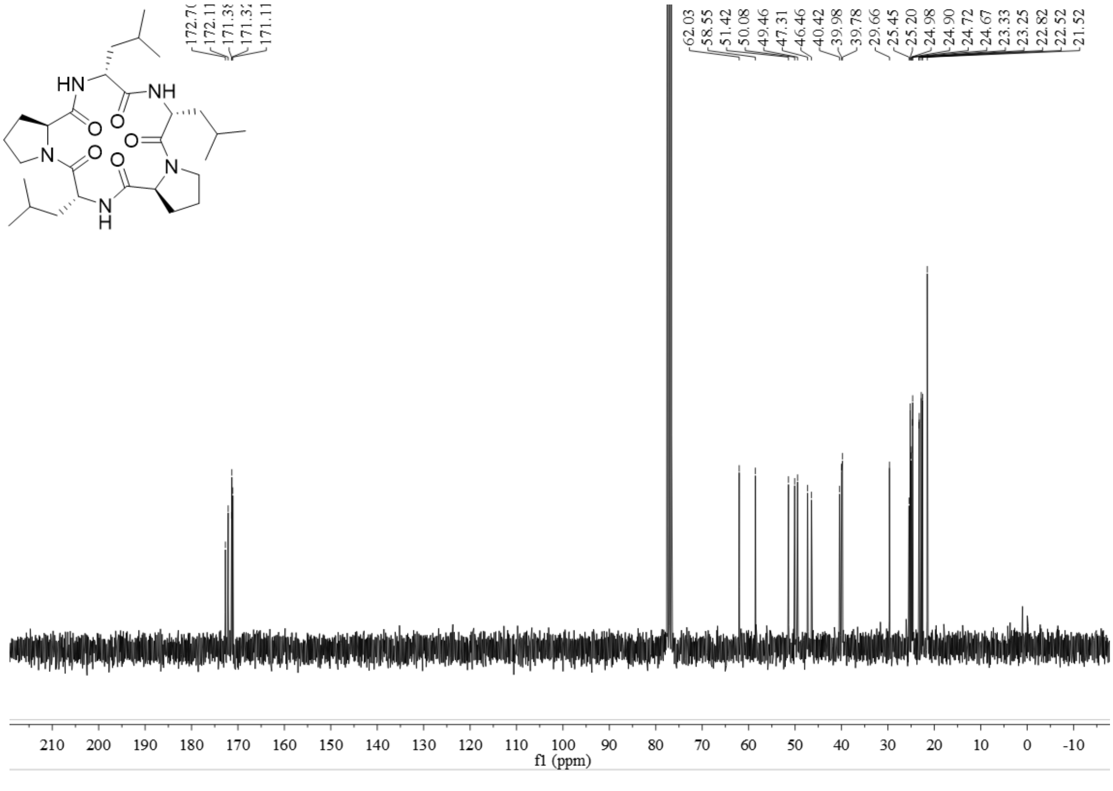


**Figure S24**. $^{13}$C NMR spectrum of **Z-8**
